# Clinical, genetic, neuroimaging, and severity spectrum of peroxisomal disorders in Iran: a multicenter cohort study

**DOI:** 10.1186/s13023-026-04549-2

**Published:** 2026-08-12

**Authors:** Golazin Shahbodagh Khan, Narges Mashayekhi, Shiva Bayat, Mehran Beiraghi Toosi, Sareh Hosseinpour, Neda Pak, Danielle Dircks, Zahra Rezaei, Reza Shervin Badv, Gholam Reza Zamani, Mahmoud Mohammadi, Mojtaba Movahednia, Parvaneh Karimzadeh, Reza Maroofian, Michael C. Kruer, Ehsan Ghayoor Karimiani, Masoud Garshasbi, Mahmoud Reza Ashrafi, Morteza Heidari, Ali Reza Tavasoli

**Affiliations:** 1 Myelin Disorders Clinic, Division of Pediatric Neurology, Pediatrics Center of Excellence, Children’s Medical Center, Tehran University of Medical Sciences, Tehran, Iran; 2 Department of Pediatrics, Division of Pediatric Neurology, Hakim Children’s Hospital, Tehran University of Medical Sciences, Tehran, Iran; 3 Department of Medical Genetics, School of Medicine, Tehran University of Medical Sciences, Tehran, Iran; 4 Department of Pediatrics, Faculty of Medicine, Mashhad University of Medical Sciences, Mashhad, Iran; 5 Rare Pediatric Neurological Disease Research Center, Mashhad University of Medical Sciences, Mashhad, Iran; 6 Department of Pediatric Neurology, Vali-e-Asr Hospital, Imam Khomeini Hospital Complex, Tehran University of Medical Sciences, Tehran, Iran; 7 Advanced Diagnostic and Interventional Radiology Research Center, Tehran University of Medical Sciences, Tehran, Iran; 8 Department of Radiology, Pediatrics Center of Excellence, Children’s Medical Center, Tehran, Iran; 9 Division of Neurology, Barrow Neurological Institute at Phoenix Children’s Hospital, Arizona, USA; 10 Division of Pediatric Neurology, Pediatrics Center of Excellence, Children’s Medical Center, Tehran University of Medical Sciences, Tehran, Iran; 11 Children Growth Disorder Research Center, Shahid Sadoughi University of Medical Sciences, Yazd, Iran; 12 Pediatric Neurology Department, Shahid Beheshti University of Medical Sciences, Tehran, Iran; 13 Department of Neuromuscular Disorders, UCL Queen Square Institute of Neurology, University College London, London, UK; 14 Department of Neuromuscular Diseases, Queen Square Institute of Neurology, University College London, London, WC1N 3BG UK; 15 Molecular and Clinical Sciences Institute, St. George’s University of London, London, SW17 0RE UK; 16 Department of Medical Genetics, Faculty of Medical Sciences, Tarbiat Modares University, Tehran, Iran

**Keywords:** Zellweger spectrum disorder, Peroxisome biogenesis disorders, Neuroimaging, Genotype–phenotype correlation, Whole-exome sequencing

## Abstract

**Background:**

Peroxisomal disorders comprise a heterogeneous group of inherited metabolic diseases including peroxisome biogenesis disorders (PBDs), single-enzyme defects, and peroxisomal dynamics disorders. Data from Middle Eastern populations remain limited despite high regional consanguinity rates. We aimed to characterize the clinical, genetic, and neuroimaging spectrum of peroxisomal disorders in a multicenter Iranian cohort.

**Methods:**

We performed a retrospective multicenter study of genetically confirmed pediatric peroxisomal disorders diagnosed between 2016 and 2023 across five referral centers. Clinical, neuroimaging, and molecular data were systematically reviewed. Patients were classified as PBDs or non-PBD peroxisomal disorders, while non-classical peroxisome-associated phenotypes were analyzed separately. A pragmatic retrospective severity index was applied to assess disease burden.

**Results:**

Forty-two patients from 42 families with peroxisomal gene-associated disorders were identified, including 40 patients in the primary analysis cohort. PBDs accounted for 33 patients and non-PBD peroxisomal disorders for seven. *PEX1* was the most frequently affected gene. Novel variants accounted for 67% of disease-associated alleles, and parental consanguinity was present in 79% of families. Developmental delay or regression was universal, while visual impairment (62%), gait abnormalities (59%), hearing impairment (54%), pyramidal signs (46%), and seizures (41%) were common. Brain MRI data were available for 20 patients, including serial studies in five, demonstrating recurrent involvement of the parieto-occipital white matter, cerebellum, brainstem, and corpus callosum, with progressive neuroimaging abnormalities observed across genetically distinct disorders. Several recurrent variants raised the possibility of regional founder effects. Non-PBD peroxisomal disorders demonstrated higher median severity indices than PBDs, although the difference did not reach statistical significance.

**Conclusions:**

This study expands the clinical, genetic, and neuroimaging spectrum of peroxisomal disorders in the Middle East and highlights extensive allelic heterogeneity, a high burden of novel variants, many occurring in the homozygous state, and substantial genotype-phenotype variability. These findings support broad genomic testing and integrated neuroimaging assessment in highly consanguineous populations.

**Supplementary Information:**

The online version contains supplementary material available at https://doi.org/10.1186/s13023-026-04549-2.

## Introduction

Peroxisomal disorders are a clinically and genetically heterogeneous group of inherited metabolic diseases broadly classified into peroxisome biogenesis disorders (PBDs), non-PBD peroxisomal disorders including single-enzyme defects and peroxisomal dynamics disorders, and a small number of non-classical peroxisome-associated phenotypes [1]. The clinical spectrum ranges from severe neonatal Zellweger syndrome to attenuated phenotypes such as Heimler syndrome [1]. Peroxisomes are essential cellular organelles involved in very-long-chain fatty acid β-oxidation, plasmalogen biosynthesis, reactive oxygen species metabolism, and neuronal development. Consequently, peroxisomal dysfunction may affect multiple organ systems, particularly the central nervous system, liver, retina, and auditory pathways, resulting in broad phenotypic heterogeneity. Single-enzyme defects - including D-bifunctional protein deficiency (*HSD17B4*) and acyl-CoA oxidase 1 deficiency (*ACOX1*) - share overlapping neurological features with PBDs but arise from distinct mechanisms and may present diagnostic challenges when biochemical markers are normal [1, 2]. *PEX1* variants account for nearly 70% of Zellweger spectrum disorder (ZSD) cases in Western cohorts, and genotype–phenotype correlations suggest that missense variants are generally associated with milder phenotypes, whereas truncating variants produce severe neonatal presentations [2, 3].

The vast majority of published cohorts originate from Western European and North American centers, leaving populations with high consanguinity rates substantially underrepresented. In the Middle East and North Africa (MENA) region, where consanguinity rates range from 20% to over 50%, a growing but still limited number of cohort studies have begun to address this gap. Al-Essa et al. reported 11 Saudi patients with Zellweger syndrome, noting population-specific features including the absence of renal cysts [4]. Alshenaifi et al. described the largest Arab cohort to date (72 families), identifying long-term survivors and patients with normal very-long-chain fatty acid (VLCFA) levels [5]. Zaki et al. delineated a founder *PEX12* variant in 20 Egyptian patients with a uniformly severe phenotype and the distinctive feature of sparse hair [6]. Zaabi et al. reported an atypical *PEX16*-related PBD in consanguineous UAE kindred with mild biochemical disruptions and prolonged survival, highlighting the diagnostic challenge of subtle VLCFA elevations [7]. Havali et al. described two Turkish patients with severe Zellweger syndrome caused by novel missense variants in *PEX1* and *PEX10*, arguing against a simple genotype–phenotype correlation for missense variants [8]. Most recently, AlMoallem et al. reported the first Saudi cohort of Heimler syndrome caused by novel *PEX6* variants, initially misdiagnosed as Usher syndrome [9]. These studies collectively highlight the potential for population-specific founder variants, atypical presentations, and diagnostic pitfalls in consanguineous MENA populations.

Iran, with a consanguinity rate of approximately 37% and a population exceeding 90 million, represents one of the largest and most genetically diverse populations in the MENA region. Ashrafi et al. reported the highest annual incidence of childhood leukodystrophies worldwide (3.01 per 100,000) based on the Iranian neurometabolic bioregistry system, attributing this in part to the high rate of consanguineous marriages [10]. The same group subsequently established the Iranian Leukodystrophy Registry and reported on 245 diagnosed patients from 680 registrants, identifying causal variants in 110 genes with 78 novel variants; peroxisomal disorders accounted for 6.5% of diagnoses, with X-linked adrenoleukodystrophy being the most common subtype [11]. Mahdieh et al. independently evaluated 152 Iranian children with leukodystrophies, achieving a 75% diagnostic rate and identifying 38 novel variants across 35 different leukodystrophy types [12]. These studies established the epidemiological and genetic framework for white matter disorders in Iran but did not provide detailed phenotypic, neuroimaging, or genotype–phenotype characterization of the peroxisomal subgroup. Despite increasing implementation of genomic testing across the MENA region, detailed peroxisomal cohort studies integrating neuroimaging evolution, genotype–phenotype analysis, and variant interpretation remain limited. This knowledge gap is particularly important in highly consanguineous populations, where rare homozygous and population-specific variants may be substantially underrepresented in publicly available genomic databases.

Most of these cohorts focus exclusively on PBDs or individual *PEX* genes, with few comparing PBDs and single-enzyme defects within the same cohort [4–7]. Also, standardized severity tools such as the Klouwer ZSD score were designed for prospective PBD-only settings and may not be applicable to retrospective multicenter cohorts including both disorder categories [13]. Here, we present the first multicenter Iranian cohort of 42 genetically confirmed patients from 42 individual families with peroxisomal disorders, encompassing PBDs spanning the full Zellweger spectrum and single-enzyme defects. We describe the clinical and genetic spectrum, characterize neuroimaging patterns, and propose a pragmatic clinical severity classification designed for retrospective and resource-limited settings.

## Methods

### Study design and participants

This retrospective multicenter cohort study was conducted between 2016 and 2023 across five centers: the Myelin Disorders Clinic at Tehran University of Medical Sciences (coordinating center); two neurogenetic centers at Mashhad University of Medical Sciences; the Molecular and Clinical Sciences Institute at St George’s, University of London; Department of Neurology, Mofid Hospital, Shahid Beheshti University of Medical Sciences; and the Department of Medical Genetics, Tarbiat Modares University, Tehran (Supplementary Fig. 1).

Patients were eligible if they had genetic confirmation of a pathogenic, likely pathogenic, or variant of uncertain significance (VUS) in a peroxisomal disorder-associated gene identified by whole-exome sequencing (WES) or targeted single-gene testing, were younger than 20 years at diagnosis, and had clinical and/or neuroimaging data sufficient for phenotypic characterization. Written informed consent was obtained from a parent or legal guardian. X-linked adrenoleukodystrophy (*ABCD1*-related) was excluded given the extensive existing literature; 27 ALD patients identified during the study period were not included. The geographic distribution of participating Iranian referral centers is illustrated in Supplementary Fig. 2.

### Classification of peroxisomal disorders

The primary analysis cohort comprised classical peroxisomal disorders with established peroxisomal dysfunction. Patients were classified into two groups: (i) peroxisome biogenesis disorders caused by pathogenic variants in *PEX* genes, including the full phenotypic spectrum of ZSDs, rhizomelic chondrodysplasia punctata type 1 (RCDP1), infantile Refsum disease, and Heimler syndrome; and (ii) non-PBD peroxisomal disorders, including *ACOX1*-related acyl-CoA oxidase deficiency, *HSD17B4*-related D-bifunctional protein deficiency, and *MFF*-related peroxisomal fission/dynamics disorder. Two patients with non-classical peroxisome-associated phenotypes were analyzed separately: Mitchell syndrome due to a heterozygous gain-of-function variant in *ACOX1* (*n* = 1) and Perrault syndrome type 1 associated with biallelic *HSD17B4*variants (*n* = 1). These patients were retained in genetic summaries but excluded from comparative clinical and neuroimaging analyses because their disease mechanisms differ substantially from classical peroxisomal disorders.

### Clinical data collection

Clinical and demographic data were extracted from registry databases and medical records. Variables included age, sex, age at symptom onset, parental consanguinity, family history, and clinical features including developmental delay or regression, seizures, visual and auditory impairments, hypotonia, pyramidal signs, movement disorders, and gait abnormalities. All data were anonymized and entered a shared structured database. Genetic diagnoses and phenotypic classifications were confirmed through joint review by genetic counselors and pediatric neurologists at each site.

### Neuroimaging evaluation

All available brain magnetic resonance imaging (MRI) studies were independently reviewed by experienced pediatric neurologists and neuroradiologists at each center, with discrepancies resolved through joint virtual consensus meetings. MRI abnormalities were classified based on established leukodystrophy patterns, including delayed myelination, hypomyelination, demyelinating changes, and cerebral and cerebellar atrophy. Particular attention was given to regions commonly affected in peroxisomal disorders: parieto-occipital white matter, corpus callosum, brainstem, cerebellar white matter including the hilus of the dentate nucleus, and middle cerebellar peduncles [14–16]. MRI severity was assigned using predefined study criteria based on the extent of white matter involvement and major structural brain abnormalities, to ensurr consistent application of the scoring framework across participating centers.

### Genetic analysis

Genomic DNA was extracted from peripheral blood using standard protocols. WES was performed on a NovaSeq 6000 platform (Illumina) with average coverage depth of 100×. Raw data were processed through an in-house bioinformatics pipeline including alignment to GRCh38/hg38, variant calling, and annotation. Variant filtration used minor allele frequency data from gnomAD and ExAC databases. Prioritization was based on predicted functional impact, segregation analysis where available, and consistency with the patient’s clinical and neuroimaging phenotype. Frameshift, nonsense, and canonical splice-site variants ranked highest. Final classification followed American College of Medical Genetics and Genomics (ACMG) 2015 guidelines, integrating molecular data with clinical and radiological findings [17]. Segregation analysis was performed by Sanger sequencing in probands and available parents. Variants classified as VUS were retained only when the overall clinical, neuroimaging, and genetic evidence strongly supported disease causality. In addition to ACMG classification, variant interpretation incorporated phenotypic concordance with the known disease spectrum, segregation analysis where parental samples were available, rarity or absence in population databases, predicted functional impact, and multidisciplinary review by experienced clinical geneticists and pediatric neurologists. Because functional studies were unavailable for most novel variants, these variants were conservatively retained as VUS according to ACMG criteria despite supportive clinical evidence.

### Clinical severity classification

Although a validated 14-domain severity score has been developed for prospective assessment of Zellweger spectrum disorders, it was designed specifically for ZSD and incorporates numerous multisystem variables (including craniofacial, skeletal, gastrointestinal, renal, endocrine, and other systemic manifestations) that were not consistently available in our retrospective multicenter cohort [13]. A pragmatic retrospective clinical severity classification was applied based on six domains: age at onset (> 12 months = 0; 3–12 months = 1; ≤3 months = 2), developmental outcome (mild delay = 0; moderate = 1; profound delay/regression = 2), seizures (none = 0; controlled = 1; refractory = 2), feeding/respiratory support (none = 0; temporary = 1; long-term = 2), disease course (stable = 0; slowly progressive = 1; rapidly progressive/early death = 2), and neuroimaging severity (normal = 0; non-specific or mild abnormalities = 1; major structural or extensive abnormalities = 2). Total scores ranged from 0 to 12. As MRI data were unavailable for some patients, an adjusted severity index was calculated for all patients by dividing the observed cumulative score by the maximum possible score based on available other five domains, thereby normalizing severity assessment across patients with complete and incomplete datasets. In patients without MRI data, the neuroimaging domain was excluded from both the numerator and denominator calculations. Overall severity was categorized as mild (≤ 0.25), moderate (0.26–0.50), or severe (> 0.50). Therefore, we adapted its conceptual framework to develop a simplified neurological severity index using six domains that were consistently documented across participating centers, clinically meaningful, and applicable across both peroxisome biogenesis disorders and non-PBD peroxisomal disorders. The resulting index was intended as a descriptive, hypothesis-generating tool for retrospective cohort-level phenotypic stratification rather than a validated prognostic or outcome measure.

### Statistical analysis

Statistical analyses were performed using IBM SPSS Statistics version 29 (IBM Corp., Armonk, NY, USA). Continuous variables were summarized using medians and interquartile ranges (IQRs), while categorical variables were reported as frequencies and percentages. Comparisons between two independent groups were performed using the Mann–Whitney U test. Comparisons across more than two independent groups were assessed using the Kruskal–Wallis test. Categorical variables were compared using Fisher’s exact test or the chi-square test, as appropriate. All statistical tests were two-sided, and a p-value < 0.05 was considered statistically significant. Given the exploratory nature of the study and the rarity of peroxisomal disorders, no adjustments were made for multiple comparisons. Accordingly, subgroup analyses were considered exploratory and interpreted cautiously because of the limited sample sizes inherent to these rare disorders. Statistical analyses were performed on the primary analysis cohort, excluding patients with non-classical peroxisome-associated phenotypes unless otherwise specified.

## Results

### Cohort demographics and classification

**Fig. 1:**
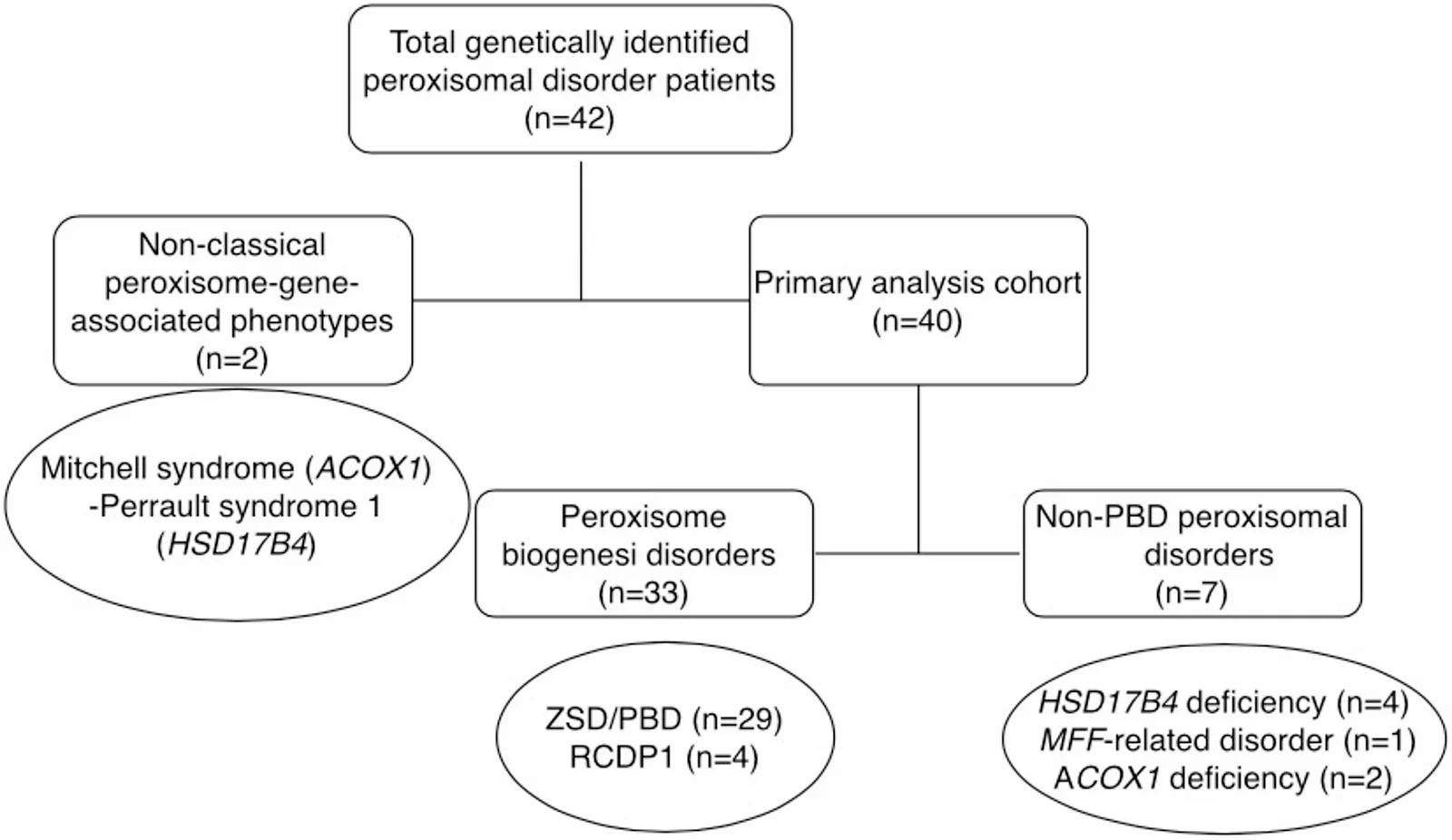
Cohort flow diagram. Classification of 42 genetically confirmed patients with peroxisomal disorder-associated gene variants. Two patients with non-classical phenotypes were analyzed separately, leaving a primary analysis cohort of 40 patients (33 PBDs and 7 non-PBD peroxisomal disorders)

A total of 42 patients from 42 families with genetically confirmed variants in peroxisomal disorder-associated genes were enrolled across five centers. Two patients with non-classical peroxisome-associated phenotypes-one with Mitchell syndrome due to a heterozygous gain-of-function *ACOX1* variant and one with Perrault syndrome type 1 associated with biallelic *HSD17B4* variants-were analyzed separately. The primary analysis cohort therefore comprised 40 patients. Among the primary analysis cohort, 33 patients had PBDs, including 29 with Zellweger spectrum disorders and four with rhizomelic chondrodysplasia punctata type 1. Seven patients had non-PBD peroxisomal disorders, including four with *HSD17B4*-related D-bifunctional protein deficiency, two with *ACOX1*deficiency, and one with an *MFF*-related peroxisomal fission/dynamics disorder. The male-to-female ratio was 24:18 (57% male). Parental consanguinity was documented in 33 of 42 families (79%), and nearly all pathogenic or likely pathogenic variants occurred in the homozygous state, consistent with the high rate of consanguinity within the cohort. The cohort flow is summarized in Fig. 1.

### Genetic spectrum

Disease-associated variants classified as pathogenic, likely pathogenic, or VUS with strong clinical and genetic evidence were identified in 13 distinct peroxisomal disorder-associated genes (Table 1). Among patients with peroxisome biogenesis disorders, *PEX1* was the most frequently affected gene (*n* = 10, 25% of the primary analysis cohort), followed by *PEX7* (*n* = 4, 10%), *PEX13* (*n* = 3, 8%), *PEX12* (*n* = 3, 8%), *PEX16* (*n* = 3, 8%), *PEX10* (*n* = 2, 5%), *PEX11B* (*n* = 2, 5%), *PEX3* (*n* = 2, 5%), *PEX6* (*n* = 2, 5%), *PEX26* (*n* = 1, 3%), and *PEX5* (*n* = 1, in the digenic *PEX5/PEX12* case). Among non-PBD peroxisomal disorders, *HSD17B4* variants were identified in four patients (10%), *ACOX1* variants in two (5%), and an *MFF* variant in one patient (3%).

**Table 1:** Clinical and genetic characteristics of 42 patients with classical peroxisomal disorders included in the primary analysis cohort

Patient	Sex	Gene	Variant	Variant ID(*p*)	ZYGOSITY	ACMG	Age of Onset	Clinical Diagnosis	Major neurological features	Survival Status
P1	M	*ACOX1*	c.679G > A	p.Gly227Ser	Hom	VUS	3 m	Peroxisomal acyl-CoA oxidase deficiency	NDD/regression, vision problems, pyramidal sign, seizures, hypotonia, movement disorder, gait disturbance, hearing impairment	Yes
P2	F	*ACOX1*	c.908dup	p.Tyr303Ter	Hom	LP	2y	Peroxisomal acyl-CoA oxidase deficiency	NDD/regression, pyramidal sign, seizures, hypotonia, gait disturbance,	Yes
P3	M	*HSD17B4*	c.1415_1417d el	p.Ser472del	Hom	VUS	3.5y	D-Bifunctional Protein Deficiency	NDD/regression, vision problems, pyramidal signs, seizures, hypotonia, movement disorder, gait disturbance, hearing impairment	No
P4	F	*HSD17B4*	c.43 A > G	p.Thr15Ala	Hom	VUS	1 m	D-Bifunctional Protein Deficiency	NDD/regression, pyramidal sign, seizures, hypotonia, gait disturbance,	Yes
P5	M	*HSD17B4*	c.665T > G	p.Val222Gly	Hom	VUS	5 m	D-Bifunctional Protein Deficiency	NDD/regression, pyramidal sign, hypotonia, movement disorder, gait disturbance,	Yes
P6	F	*HSD17B4*	c.1501 C > A	p.Gln501Lys	Hom	VUS	9 m	D-Bifunctional Protein Deficiency	NDD/regression, seizure	N/A
P7	M	*MFF*	c.352–2 A > C	N/A	Hom	LP	1.5y	Peroxisomal fission/dynamic disorders	NDD/regression, pyramidal sign, seizures, hypotonia, movement disorder, gait disturbance,	N/A
P8	F	*PEX1*	c.2528G > A	p.Gly843Asp	Hom	P	7 m	ZSD/PBD	NDD/regression, vision problems, hypotonia, gait disturbance, hearing impairment	No
P9	M	*PEX1*	c.1706 A > C	p.His569Pro	Hom	VUS	13y	ZSD/PBD	NDD/regression, vision problems, hearing impairment	Yes
P10	M	PEX1	c.2357G > A	*p*.Gly843Asp	Hom	*P*	4y	ZSD/PBD	NDD/regression, vision problems, hypotonia, gait disturbance, hearing impairment	Yes
P11	M	*PEX1*	c.G3550T	p.Gly1184Cys	Hom	LP	1 m	ZSD/PBD	NDD/regression, vision problems, pyramidal sign, seizures, hypotonia, movement disorder, gait disturbance	Yes
P12	M	*PEX1*	c.C2945T	p.Thr982Ile	Hom	VUS	6 m	ZSD/PBD	NDD/regression, vision problem, hypotonia, gait disturbance	Yes
P13	F	*PEX1*	c.A1G	p.Met1	Hom	VUS	1 m	ZSD/PBD	NDD/regression, seizures, hypotonia, gait disturbance	Yes
P14	F	*PEX1*	c.2528G > A	p.Gly843Asp+L2 8:L32	Hom	P	1 m	ZSD/PBD	NDD/regression, pyramidal sign, hearing impairment	Yes
P15	F	*PEX1*	c.2528G > A	p.Gly843Asp	Hom	P	1 m	ZSD/PBD	NDD/regression, pyramidal sign, hearing impairment	Yes
P16	M	*PEX1*	c.3116 C > T	p.Thr1039Ile	Hom	VUS	N/A	ZSD/PBD	NDD/regression, vision problems, hypotonia, movement disorder, hearing impairment	No
P17	M	*PEX1*	c.2357G > A	p.Gly786Asp	Hom	P	4y	ZSD/PBD	NDD/regression, vision problems, hearing impairment	Yes
P18	M	*PEX10*	c.98T > G	p.Leu33Arg	Hom	LP	13 m	ZSD/PBD	NDD/regression, vision problems, pyramidal sign, hypotonia, gait disturbance, hearing problems	No
P19	M	*PEX10*	c.98T > G	p.Leu33Arg	Hom	LP	12 m	ZSD/PBD	NDD/regression, vision problems, pyramidal sign, hypotonia, movement disorder, gait disturbance, hearing impairment	No
P20	F	*PEX11B*	c.136 C > T	p.Arg46*	Hom	LP	1 m	ZSD/PBD	NDD/regression, vision problems, seizures, hypotonia, gait disturbance	Yes
P21	M	PEX11B	c.524del	*p*.Gly175AspfsTe r14	Hom	LP	1 m	ZSD/PBD	NDD/regression, vision problems, pyramidal sign, gait disturbance	Yes
P22	M	*PEX12*	c.430T > C	p.Ser144Pro	Hom	VUS	8y	ZSD/PBD	NDD/regression, vision problems, pyramidal sign, movement disorder, gait disturbance, hearing impairment	Yes
P23	M	*PEX12*	c.280dup	p.Met94Asnfs*1 2	Hom	LP	congenital	ZSD/PBD	NDD/regression, vision problems, seizures, hypotonia, gait disturbance	Yes
P24	M	*PEX12*	c.280dup	p.Met94Asnfs*12	Hom	LP	N/A	ZSD/PBD	NDD/regression, seizure, hypotonia	N/A
P25	M	*PEX13*	c.938G > A	p.Trp313*	Hom	LP	N/A	ZSD/PBD	NDD/regression, seizure, hypotonia, movement disorder	No
P26	F	*PEX13*	c.970G > C	p.Gly324Arg	Hom	VUS	N/A	ZSD/PBD	NDD/regression, vision problems, movement disturbance	No
P27	M	*PEX13*	c.529_531del	p.Lys177del	Hom	VUS	congenital	ZSD/PBD	NDD/regression, seizures, hypotonia, gait disturbance	No
P28	M	*PEX16*	c.C859T	p.Arg287Cys	Hom	LP	25y	ZSD/PBD	NDD/regression, pyramidal sign, movement disorder, gait disturbance, hearing impairment	N/A
P29	M	*PEX16*	c.863 C > T	p.Ser288Phe	Hom	VUS	N/A	ZSD/PBD	Movement disorder, gait disturbance	N/A
P30	F	*PEX16*	c.829 C > T	p.Arg277Trp	Hom	LP	N/A	ZSD/PBD	NDD/regression, vision problems, seizures, movement disorder, gait disturbance	N/A
P31	M	*PEX26*	c.794_795insC CTT	p.Val266LeufsTe r6	Hom	LP	congenital	ZSD/PBD	NDD/regression, hypotonia, gait disturbance, hearing impairment	Yes
P32	M	*PEX3*	N/A	p.Gln62Lys	Hom	VUS	6y	ZSD/PBD	NDD/regression, pyramidal sign, movement disorder, gait disturbance	Yes
P33	F	*PEX3*	c.619 C > T	p.Gly217*	Hom	LP	congenital	ZSD/PBD	NDD/regression, seizures, hypotonia, gait disturbance,	Yes
P34	F	*PEX5*,* PEX12*	c.137_147 + 1d el, c.541T > G	No, p.Tyr181Asp	Hom, Hom	LP, LP	2y	ZSD/PBD	NDD/regression, pyramidal signs, hypotonia, movement disorder, gait disturbance	Yes
P35	M	*PEX6*	c.231 C > A	p.Ser77Ar	Hom	VUS	7 m	ZSD/PBD	NDD/regression, vision problems, seizures, gait disturbance, haring problems	Yes
P36	F	*PEX6*	c1992G > C	p.Glu664Asp	Hom	LP	congenital	ZSD/PBD	NDD/regression, seizures, hypotonia, hearing impairment	Yes
P37	F	*PEX7*	c.370_396del	p.Gly124_Ser13 2del	Hom	VUS	12 m	RCDP type 1/PBD	NDD/regression, pyramidal sign, hypotonia, movement disorder, gait disturbance	Yes
P38	F	PEX7	c.209T > G	(*p*.Leu70Trp)	Hom	LP	*N*/A	RCDP type 1/PBD	NDD/regression, vision problems	*N*/A
P39	F	*PEX7*	c.370_395del	p.Gly124Metfs*2 0	Hom	LP	N/A	RCDP type 1/PBD	NDD/regression	N/A
P40	F	*PEX7*	c.884G > C	p.Ser295Thr	Hom	VUS	N/A	RCDP type 1/PBD	NDD/regression, seizures	No
P41	M	*ACOX1*	c.3G > A	p.Met1?	Het	VUS	1y	Mitchell Syndrome	NDD/regression, hypotonia, movement disorder, gait disturbance	Yes
P42	F	*HSD17B4*	c.614T > C	(p.Met205Thr)	Hom	VUS	N/A	Perrault syndrome 1	NDD/regression, vision problems, hypotonia, movement disorder, gait disturbance, hearing impairment	N/A

Abbreviations: ACMG, American College of Medical Genetics and Genomics; DD/ID, developmental delay/intellectual disability; LP, likely pathogenic; NDD: Neurodevelopmental delay; P, pathogenic;

RCDP: Rhizomelic chondrodysplasia punctata; VUS, variant of uncertain significance. Severity index was normalized for missing neuroimaging data as described in the Methods section

Variant types included missense variants (*n* = 24 alleles, 57%), frameshift variants (*n* = 5, 12%), in-frame deletions (*n* = 5, 12%), nonsense variants (*n* = 4, 10%), splice-site variants (*n* = 2, 5%), and start-loss variants (*n* = 2, 5%). ACMG classifications were pathogenic in five patients, likely pathogenic in 17, and variants of uncertain significance (VUS) in 17, reflecting the large proportion of rare and previously unreported variants in this understudied population. Novel variants, defined as absent from ClinVar and previously unreported in the literature, accounted for 67% of disease-associated alleles. Segregation analysis by Sanger sequencing confirmed inheritance patterns in all families for whom parental DNA was available. The majority of pathogenic or likely pathogenic variants were identified in the homozygous state, reflecting the high prevalence of parental consanguinity within the study population. Several genes demonstrated substantial intragenic phenotypic heterogeneity. Patients harboring variants in *PEX1* and *PEX13* showed particularly broad clinical severity ranges, spanning mild Heimler syndrome phenotypes to severe neonatal presentations with early mortality.

### Recurrent, shared, and notable variants

The *PEX1* p.Gly843Asp variant, the most common *PEX1* allele reported worldwide, was identified in three unrelated families. All patients were homozygous for the variant and generally exhibited milder phenotypes, including Heimler syndrome or intermediate ZSD presentations. However, one patient developed severe progressive disease and died early in life despite carrying this traditionally hypomorphic allele. The *PEX1* p.Gly786Asp variant was identified in two unrelated patients, both presenting with Heimler syndrome. The *PEX10* p.Leu33Arg variant was identified in two affected individuals from the same family, both of whom developed developmental regression following infection, rapid neurological deterioration, and early mortality. Serial MRI studies demonstrated progression from focal demyelination to diffuse cerebral white matter and brainstem involvement. The *PEX16* p.Arg287Cys variant identified in one patient was previously reported in a consanguineous family from the United Arab Emirates with atypical PBD and prolonged survival. Similarly, the *PEX12* p.Met94Asnfs*12 frameshift variant was identified in two unrelated Iranian patients. One patient harbored homozygous variants in both *PEX5* (splice-site) and *PEX12* (p.Tyr181Asp). Despite dual *PEX* gene involvement, the patient demonstrated a relatively mild phenotype characterized by developmental regression at 2 years of age, hearing impairment, and an adjusted severity index of 0.25. Although these recurrent variants may reflect regional enrichment, no conclusions regarding founder effects can be drawn because haplotype analysis was not performed.

### Clinical features

Clinical data were available for all 40 patients in the primary analysis cohort. Neurodevelopmental delay or regression was universal (40/40, 100%). Other frequent clinical manifestations included vision abnormalities, gait disturbance, hearing impairment, pyramidal signs, seizures, hypotonia, and movement disorders. Microcephaly was documented in approximately one-quarter of patients.

The most common initial presentation was global developmental delay, followed by developmental regression and congenital hypotonia with seizures. Several patients initially presented with isolated sensory deficits or gait abnormalities before developing more diffuse neurological dysfunction. Age at symptom onset was available for 35 patients. Congenital or neonatal onset (≤ 3 months) occurred in 16 patients (46%), onset between 3 and 12 months in 9 patients (26%), and onset after 12 months in 10 patients (29%). Mortality occurred in seven patients, all within the PBD subgroup and all associated with severe phenotypes (adjusted severity index ≥ 0.58). Although hepatopathy and dysmorphic features were not systematically collected across all centers, cholestatic liver disease was observed predominantly among patients with severe neonatal presentations.

Two patients with non-classical peroxisome-associated phenotypes were analyzed separately. A patient with Mitchell syndrome carried a heterozygous novel start-loss variant in *ACOX1* (c.3G > A, p.Met1?) and presented at 1 year of age with developmental delay, hypotonia, movement disorder, gait disturbance, and microcephaly. A second patient with Perrault syndrome type 1 harbored a homozygous novel *HSD17B4* missense variant (c.614T > C, p.Met205Thr) and exhibited developmental delay, visual impairment, hearing impairment, hypotonia, movement disorder, and gait abnormalities. These patients were excluded from comparative analyses because of their distinct disease mechanisms.

### Neuroimaging findings

**Fig. 2:**
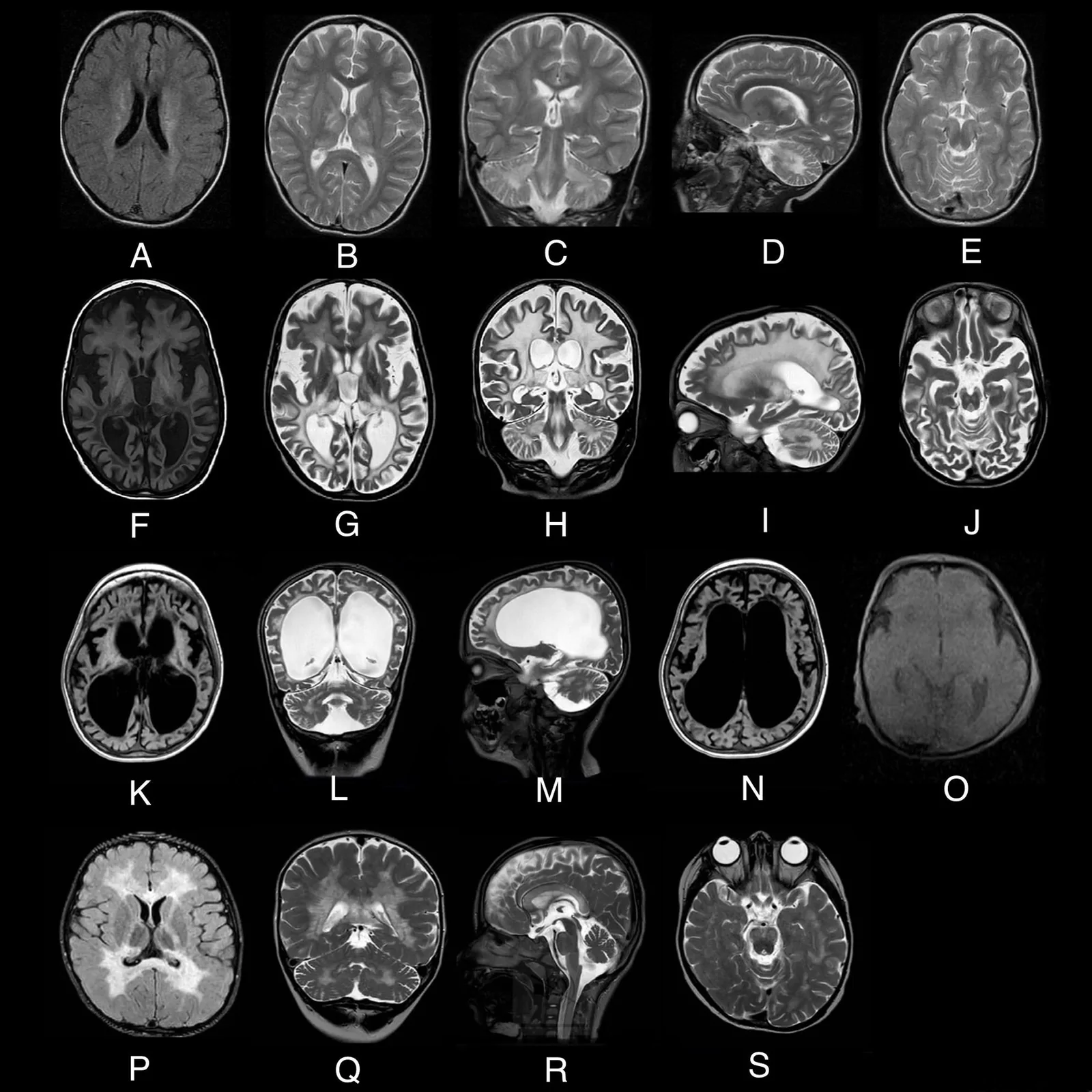
Representative neuroimaging findings across the spectrum of peroxisomal disorders. **A**–**J**: Serial MRI studies in a patient with *HSD17B4*-related D-bifunctional protein deficiency (c.1415-1417del variant) at 3.5 years (**A**–**E**) and 6 years (**F**–**J**). Axial FLAIR and T2-weighted images demonstrate progressive diffuse deep and parieto-occipital white matter abnormalities with thalamic involvement. Coronal and sagittal T2-weighted sequences show progressive involvement of the brainstem, cerebellar white matter, and hilus of the dentate nucleus, accompanied by increasing cerebral atrophy. Axial T2-weighted images demonstrate involvement of the cerebral peduncles, red nuclei, and periaqueductal gray matter. **K**–**N**: Patient with *ACOX1* deficiency (c.908dup variant) showing diffuse cerebral and cerebellar white matter and dentate hilus involvement, cerebral and basal ganglia atrophy, ventriculomegaly, and polymicrogyria. **O**: Axial T1-weighted image demonstrating frontal polymicrogyria in a patient with *PEX12*-related PBD (c.280dup variant). **P**–**S**: Patient with *PEX5*-related PBD (c.137–147 + 1 del, c.541T > G variant). Axial FLAIR and T2-weighted images demonstrate symmetric confluent cerebral white matter abnormalities involving the corpus callosum, thalami, internal capsules, and pyramidal tracts. Coronal and sagittal T2-weighted sequences show cerebellar white matter and dentate hilus involvement with extension into the brainstem pathways

Brain MRI studies were available for 25 imaging time points representing 20 patients in the primary analysis cohort, including serial imaging in five patients. Nineteen imaging studies demonstrated abnormal findings (Fig. 2).

The most frequent MRI abnormalities included demyelinating white matter changes (68%), brainstem involvement including pontine and midbrain abnormalities (60%), cerebellar involvement affecting the dentate hilus, cerebellar white matter, or middle cerebellar peduncles (56%), deep white matter involvement (52%), parieto-occipital predominance (40%), and corpus callosum abnormalities or thinning (40%). Additional findings included delayed myelination, cerebellar atrophy, cerebral atrophy, cortical dysplasia/polymicrogyria, and contrast enhancement. Six patients demonstrated normal or near-normal MRI studies; all belonged to the milder clinical subgroup with low severity indices. Detailed neuroimaging findings stratified by gene and severity category are summarized in Table 2. Structural cortical abnormalities, including polymicrogyria, were identified in three patients with variants in *PEX1*, *PEX12*, and *ACOX1*. All demonstrated congenital onset and severe neurological phenotypes. Neuroimaging abnormalities frequently followed recognizable anatomical progression patterns. Early disease commonly involved the cerebellar hilus and parieto-occipital white matter before extending into diffuse cerebral white matter and brainstem structures in more advanced stages.

**Table 2:** Severity scoring and adjusted severity index for 39 patients with classical peroxisomal disorders included

Patient ID	Gene	Clinical Diagnosis	Variant	Age at onset Score	Developmental Score	Seizure Score	Feeding/Resp Score	Disease course score	MRI severity score	Raw Total Score	Adjusted Severity Index	Severity Category
P1	*ACOX1*	Peroxisomal acyl-CoA oxidase-def	c.679G > A	1	1	1	0	1	2	6	0.5	Severe
P2	*ACOX1*	Peroxisomal acyl-CoA oxidase-def	c.908dup	0	2	1	1	2	2	8	0.66	Severe
P3	*HSD17B4*	D-Bifunctional Protein-def	c.1501 C > A	1	1	1	0	1	N/A	4	0.33	Moderate
P4	*HSD17B4*	D-Bifunctional Protein-def	c.665T > G	1	2	0	2	1	1	6	0.5	Severe
P5	*HSD17B4*	D-Bifunctional Protein-def	c.43 A > G	2	2	1	0	2	1	7	0.58	Severe
P6	*HSD17B4*	D-Bifunctional Protein-def	c.1415_141 7del	0	2	1	2	2	2	9	0.75	Severe
P7	*MFF*	Peroxisomal fission/dynamic disorders	c.352–2 A > C	0	1	1	1	2	N/A	5	0.41	Moderate
P9	*PEX1*	ZSD/PBD	c.2357G > A	0	1	0	0	1	1	3	0.25	Mild
P10	*PEX1*	ZSD/PBD	c.2528G > A	2	0	0	0	0	1	3	0.25	Mild
P11	*PEX1*	ZSD/PBD	c.2528G > A	2	0	0	0	0	1	3	0.25	Mild
P12	*PEX1*	ZSD/PBD	c.1706 A > C	0	1	0	0	1	2	4	0.33	Moderate
P13	*PEX1*	ZSD/PBD	c.A1G	2	1	1	0	0	1	4	0.33	Moderate
P14	*PEX1*	ZSD/PBD	c.C2945T	1	2	1	0	2	N/A	6	0.5	Severe
P15	*PEX1*	ZSD/PBD	c.2528G > A	1	2	0	1	2	N/A	6	0.6	Severe
P16	*PEX1*	ZSD/PBD	c.G3550T	2	2	1	2	1	1	8	0.66	severe
P17	*PEX1*	ZSD/PBD	c.2357G > A	0	0	0	0	0	N/A	0	0	mild
P18	*PEX10*	ZSD/PBD	c.98T > G	0	2	0	1	2	2	7	0.58	severe
P19	*PEX10*	ZSD/PBD	c.98T > G	1	2	0	1	2	2	8	0.66	Severe
P20	*PEX11B*	ZSD/PBD	c.524del	2	0	0	0	0	1	3	0.25	Mild
P21	*PEX11B*	ZSD/PBD	c.136 C > T	2	1	1	0	0	0	4	0.33	Moderate
P22	*PEX12*	ZSD/PBD	c.430T > C	0	0	0	0	0	2	2	0.16	Mild
P23	*PEX12*	ZSD/PBD	c.280dup	2	1	1	0	1	N/A	5	0.41	Moderate
P24	PEX12	ZSD/PBD	c.280dup	2	2	2	1	2	2	11	0.91	Severe
P25	*PEX13*	ZSD/PBD	c.970G > C	0	1	0	0	1	N/A	2	0.16	Mild
P26	*PEX13*	ZSD/PBD	c.938G > A	2	2	2	1	2	N/A	9	0.75	Severe
P27	*PEX13*	ZSD/PBD	c.529_531d el	2	2	1	2	2	2	11	0.91	Severe
P28	*PEX16*	ZSD/PBD	c.863 C > T	0	0	0	0	1	N/A	1	0.08	Mild
P29	*PEX16*	ZSD/PBD	c.C859T	0	1	0	0	1	N/A	2	0.16	Mild
P30	*PEX16*	ZSD/PBD	c.829 C > T	0	1	1	0	1	N/A	3	0.25	Mild
P31	*PEX26*	ZSD/PBD	c.794_795i nsCCTT	2	1	0	0	0	0	3	0.25	Mild
P32	*PEX3*	ZSD/PBD	N/A	0	1	0	0	1	0	2	0.16	Mild
P33	*PEX3*	ZSD/PBD	c.619 C > T	2	2	1	1	1	N/A	7	0.58	Severe
P34	*PEX5*, *PEX12*	ZSD/PBD	c.137_147+ 1del, c.541T > G	0	0	0	0	1	2	3	0.25	Mild
P35	*PEX6*	ZSD/PBD	c.231 C > A	1	0	1	0	0	2	4	0.33	Moderate
P36	*PEX6*	ZSD/PBD	c1992G > C	2	2	1	0	0	0	5	0.41	Moderate
P37	*PEX7*	ZSD/PBD	c.209T > G	1	1	0	0	1	N/A	3	0.25	Mild
P38	*PEX7*	RCDP typ1/ PBD	c.370_395d el	1	1	**0**	0	1	N/A	3	0.25	Mild
P39	*PEX7*	RCDP typ1/ PBD	c.370_396d el	1	1	0	0	1	2	5	0.41	Moderate
P40	*PEX7*	RCDP typ1/ PBD	c.884G > C	2	1	1	1	2	1	8	0.66	Severe

- Patient IDs correspond directly to Table 1

Abbreviations: MRI, magnetic resonance imaging; PBD, peroxisome biogenesis disorder. Severity categories: mild < = 0.25, moderate 0.26–0.50, severe > 0.50. Patient IDs correspond to Table 1 after exclusion of the three non-classical peroxisome-associated disorders

### Serial MRI observations

Five patients had serial MRI studies available for longitudinal review. One patient with a variant in *HSD17B4* demonstrated progression from isolated parieto-occipital demyelination at 42 months to diffuse cerebral white matter involvement with progressive cerebellar and cerebral atrophy by 72 months. Two patients with *PEX10* variants demonstrated rapidly progressive multifocal demyelination extending into diffuse white matter, basal ganglia, and brainstem structures over 12–16 months, paralleling rapid clinical deterioration. One patient with an *ACOX1* variant exhibited progressive cerebellar and brainstem abnormalities with increasing corpus callosum thinning between 32 and 48 months.

### Clinical severity classification

**Fig. 3:**
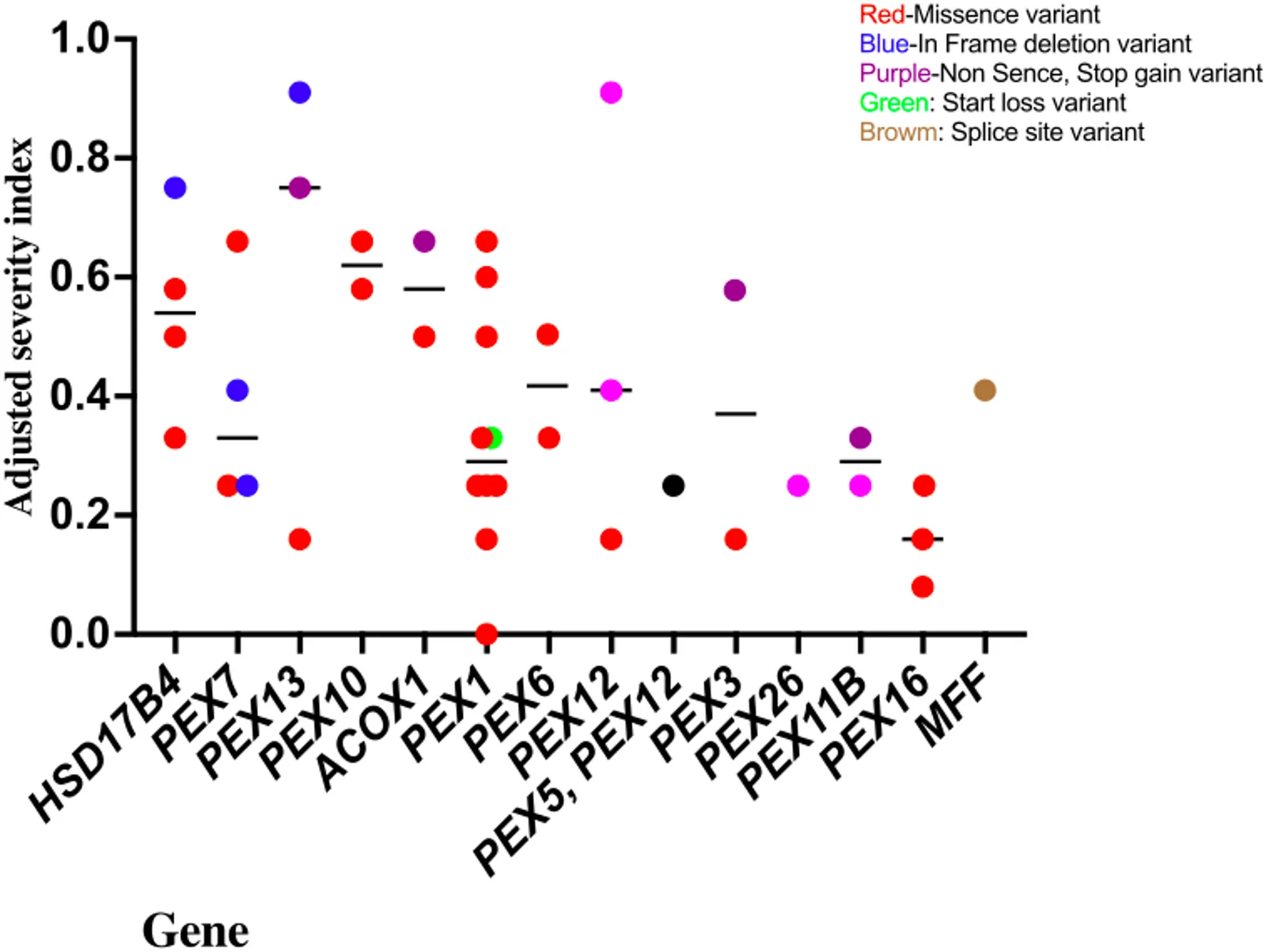
Adjusted severity index by gene and variant class in peroxisomal disorders. Each dot represents one patient. Variant types are color-coded as follows: missense (red), in-frame deletion (blue), nonsense/stop-gain (purple), start-loss (green), and splice-site (brown)

The adjusted severity index across the 40 patients in the primary analysis cohort ranged from 0.00 to 0.91 (median 0.33, IQR 0.25–0.58). Fifteen patients (38%) were classified as mild (severity index ≤ 0.25), 13 (33%) as moderate (0.26–0.50), and 12 (30%) as severe (> 0.50). Severity distribution by gene and disease subgroup is illustrated in Fig. 3. Detailed genotype, variant classification, and severity index data are summarized in Table 3.

**Table 3:** Frequency of neuroradiological abnormalities according to causative gene in patients with classical peroxisomal disorders

Gene	Patients (*n*)	Reports (*n*)	Delayed Myelination	Demyelination	Cerebellar Involvement	Structural Brain Abnormalities	Brainstem Involvement	Corpus Callosum Abnormalities	Cerebral Atrophy	Cortical Dysplasia
*ACOX1*	3	4	1/4 (25%)	3/4 (75%)	3/4 (75%)	1/4 (25%)	3/4 (75%)	1/4 (25%)	2/4 (50%)	2/4 (50%)
*HSD17B4*	2	3	2/3 (67%)	3/3 (100%)	2/3 (67%)	0/3 (0%)	3/3 (100%)	1/3 (33%)	1/3 (33%)	0/3 (0%)
*PEX1*	4	4	1/4 (25%)	1/4 (25%)	1/4 (25%)	1/4 (25%)	0/4 (0%)	2/4 (50%)	0/4 (0%)	1/4 (25%)
*PEX10*	2	3	2/3 (67%)	3/3 (100%)	3/3 (100%)	0/3 (0%)	3/3 (100%)	0/3 (0%)	0/3 (0%)	0/3 (0%)
*PEX12*	3	3	3/3 (100%)	2/3 (67%)	2/3 (67%)	0/3 (0%)	2/3 (67%)	1/3 (33%)	0/3 (0%)	1/3 (33%)
*PEX7*	2	3	1/3 (33%)	2/3 (67%)	0/3 (0%)	0/3 (0%)	2/3 (67%)	2/3 (67%)	0/3 (0%)	0/3 (0%)
*PEX13*	1	1	1/1 (100%)	1/1 (100%)	1/1 (100%)	0/1 (0%)	1/1 (100%)	1/1 (100%)	1/1 (100%)	0/1 (0%)
*PEX3*	1	1	0/1 (0%)	0/1 (0%)	0/1 (0%)	0/1 (0%)	0/1 (0%)	0/1 (0%)	0/1 (0%)	0/1 (0%)
*PEX5*,* PEX12*	1	1	0/1 (0%)	1/1 (100%)	1/1 (100%)	0/1 (0%)	0/1 (0%)	1/1 (100%)	0/1 (0%)	0/1 (0%)
*PEX6*	1	1	0/1 (0%)	0/1 (0%)	0/1 (0%)	0/1 (0%)	0/1 (0%)	0/1 (0%)	0/1 (0%)	0/1 (0%)

- **Abbreviations**: MRI, magnetic resonance imaging. Values are presented as number of affected cases/total reported cases (%)

Among *PEX1*-related disorders (*n* = 10), severity ranged from 0.00 in patients with Heimler syndrome to 0.66 in patients with severe neonatal presentations, illustrating substantial allelic and phenotypic heterogeneity. Patients carrying the recurrent *PEX1* p.Gly843Asp allele generally exhibited mild-to-moderate phenotypes, although one patient experienced severe disease progression and early mortality. Patients with *PEX13* variants demonstrated marked intragenic variability. One patient harboring *PEX13* p.Lys177del exhibited the highest severity index in the cohort (0.91) and died early in life, whereas another patient with *PEX13*p.Gly324Arg had only mild disease (severity index 0.16).

**Fig. 4:**
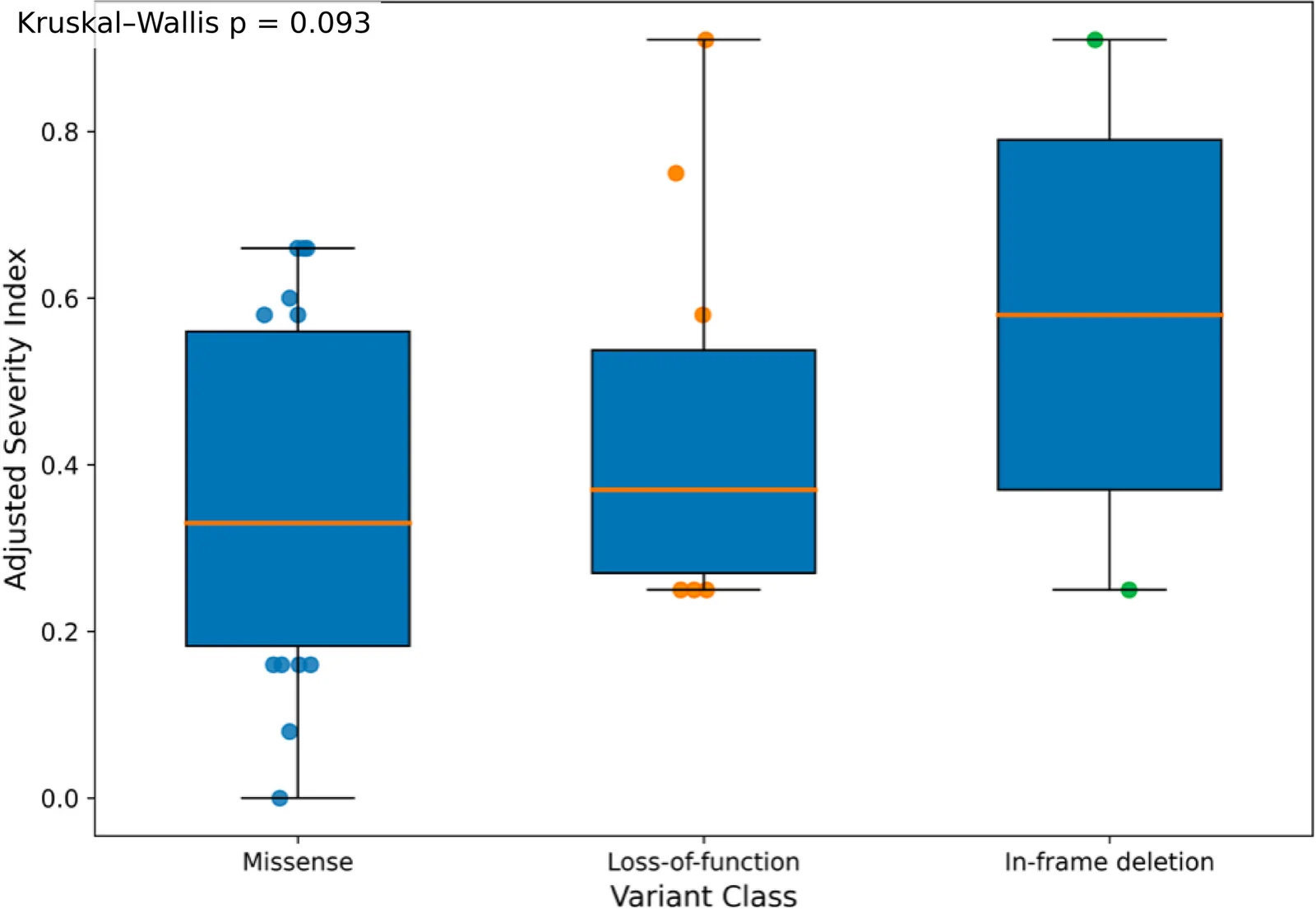
Severity index according to variant class. Distribution of adjusted severity indices across missense variants, predicted loss-of-function variants, and in-frame deletion variants. Boxes represent the interquartile range (IQR), center lines indicate medians, whiskers represent the data range, and individual data points are shown. Predicted loss-of-function variants included frameshift, nonsense, splice-site, and start-loss variants. Differences between variant classes were not statistically significant (Kruskal–Wallis *p* = 0.093)

Variant class analysis demonstrated a trend toward higher severity among patients carrying predicted loss-of-function variants (median severity index 0.41, IQR 0.33–0.64) compared with those harboring missense variants (median 0.29, IQR 0.18–0.50), although this difference did not reach statistical significance (Mann–Whitney U, *p* = 0.087). Patients with in-frame deletions also demonstrated relatively high severity indices (median 0.58), but differences between variant classes were not statistically significant (Kruskal–Wallis *p* = 0.093). Severity distribution according to variant class is illustrated in Fig. 4.

### Comparative analysis of PBDs and non-PBD peroxisomal disorders

Comparative analysis was performed between patients with PBDs (*n* = 33) and non-PBD peroxisomal disorders (*n* = 7), excluding the two patients with non-classical peroxisome-associated phenotypes. Most individual clinical manifestations did not differ significantly between groups. However, pyramidal signs and seizures were more frequent among patients with non-PBD peroxisomal disorders than among patients with PBDs (*p* = 0.029 and *p* = 0.033, respectively). Patients with PBDs demonstrated a broad clinical spectrum, ranging from mild phenotypes such as Heimler syndrome and infantile Refsum disease to severe neonatal Zellweger spectrum presentations. Mortality occurred predominantly in the PBD subgroup. The median adjusted severity index was higher in patients with non-PBD peroxisomal disorders than in patients with PBDs (0.50, IQR 0.45–0.62 vs. 0.33, IQR 0.25–0.58), although this difference did not reach statistical significance (Mann–Whitney U, *p* = 0.057). Patients with *HSD17B4*-related disease commonly presented with pyramidal signs, seizures, gait disturbance, and cerebellar involvement, whereas severe *ACOX1*-related deficiency was associated with higher severity indices. Patients with major structural or extensive MRI abnormalities (neuroimaging domain score 2) demonstrated significantly higher median adjusted severity indices than patients with normal or mild MRI findings (0.62 vs. 0.25; Mann–Whitney U, *p* < 0.01). These findings should be interpreted cautiously given the small number of patients in the non-PBD subgroup.

## Discussion

In this multicenter Iranian cohort, we describe 42 genetically confirmed patients with peroxisomal disorder-associated gene variants, including a primary analysis cohort of 40 patients. The cohort expands the clinical, genetic, and neuroimaging spectrum of peroxisomal disorders in a highly consanguineous Middle Eastern population and highlights several findings relevant to diagnosis and interpretation of rare peroxisomal variants. These include a high proportion of previously unreported variants, extensive homozygosity, recurrent or potentially founder alleles, broad intragenic phenotypic variability, and recognizable neuroimaging patterns across both PBDs and non-PBD peroxisomal disorders. An additional strength of the cohort is the inclusion of the broad spectrum of genetically confirmed peroxisomal disorders encountered in clinical practice, ranging from classical PBDs to non-PBD peroxisomal disorders and non-classical phenotypes associated with peroxisomal genes. This spectrum highlights the expanding clinical and molecular diversity of peroxisomal disease and supports a genotype-based classification approach in contemporary diagnostic practice.

The genetic architecture of this cohort differs in several important ways from large Western ZSD series. In historical datasets and international registries, *PEX1* accounts for the majority of ZSD cases, followed by *PEX6*, *PEX12*, *PEX26*, and *PEX10* [1–3, 18–20]. Although *PEX1* remained the most frequently affected gene in our cohort, accounting for 25% of patients in the primary analysis cohort, disease-causing variants were distributed across 13 distinct peroxisomal disorder-associated genes. This broader genetic distribution likely reflects the high rate of consanguinity and enrichment of rare homozygous alleles within the Iranian population. Our findings are also consistent with recent Iranian genomic studies demonstrating marked heterogeneity among inherited leukodystrophies and peroxisomal disorders [10–12]. Recently, Khalilian et al. reported additional Iranian patients with peroxisome biogenesis defects involving multiple *PEX* genes, further emphasizing the substantial allelic diversity of peroxisomal disease within this population [21].

One of the principal findings of this study is the remarkably high burden of previously unreported variants. Novel variants accounted for 67% of disease-associated alleles, and the majority of pathogenic or likely pathogenic variants were identified in the homozygous state. Together with the high prevalence of parental consanguinity, these findings underscore the substantial contribution of rare recessive variants to the burden of peroxisomal disorders in the region. They also highlight a broader challenge in rare disease genomics: populations from the Middle East remain substantially underrepresented in current reference sequencing datasets, potentially contributing to delayed diagnosis, uncertain variant interpretation, and under recognition of milder disease phenotypes. Similar concerns have recently been raised in broader genomic studies from the MENA region, where ancestry-specific variants remain inadequately represented in publicly available genomic databases [10–12, 21]. The relatively high proportion of variants classified as VUS reflects the rarity of peroxisomal disorders, the predominance of previously unreported variants, and the limited representation of Middle Eastern populations in current reference genomic databases. Many of these variants demonstrated strong concordance with the expected clinical and neuroimaging phenotype and supportive segregation findings where parental samples were available. Nevertheless, because functional validation was unavailable, we intentionally retained conservative ACMG classifications. Accordingly, genotype–phenotype correlations involving these variants should be interpreted cautiously until additional molecular, functional, or independent clinical evidence becomes available.

Several recurrent variants identified in our cohort were clinically informative. The classic hypomorphic *PEX1*p.Gly843Asp allele, one of the best-characterized *PEX1* variants worldwide, was identified in three unrelated families. Most affected individuals demonstrated mild or intermediate phenotypes, consistent with prior genotype-phenotype studies [1–3, 22]. However, one patient carrying homozygous p.Gly843Asp experienced severe disease progression and early mortality, underscoring the substantial phenotypic variability associated even with traditionally mild alleles. Recent population-genetics modeling by Malone et al. suggested that mild and intermediate *PEX1*-related phenotypes may be substantially underrecognized globally, and our findings support that possibility [23]. Similarly, the *PEX1* p.Gly786Asp variant was identified in two unrelated Iranian patients with Heimler syndrome, suggesting a potentially enriched regional allele associated with milder sensory-predominant phenotypes. In contrast, the *PEX10* p.Leu33Arg variant identified in two affected relatives was associated with rapidly progressive neurological deterioration following infection. Together with the marked neuroimaging progression observed in these patients, this finding raises the possibility that environmental or inflammatory stressors may exacerbate disease progression in partially compensated peroxisomal dysfunction.

The *PEX16* p.Arg287Cys variant identified in our cohort had previously been reported in a family from the United Arab Emirates with atypical PBD and prolonged survival, raising the possibility of a regionally enriched Middle Eastern allele [17]. Likewise, the recurrent *PEX12* p.Met94Asnfs*12 variant identified in two unrelated Iranian families may represent a recurrent regional allele and raises the possibility of a founder effect distinct from the Egyptian *PEX12* founder mutation reported by Zaki et al., although this remains speculative in the absence of haplotype analysis [6]. Because haplotype analysis was not performed, these observations should be interpreted as evidence of recurrent regional variants rather than confirmed founder effects. Future haplotype-based studies will be required to determine whether these recurrent alleles represent true founder mutations. The patient harboring homozygous variants in both *PEX5* and *PEX12* represents another notable observation. Digenic or multilocus *PEX* variation has previously been described in ZSD cohorts, and Yik et al. emphasized that complementation studies may be necessary to determine the principal disease-causing defect in such patients [24]. Despite dual *PEX* involvement, our patient demonstrated a relatively mild phenotype, suggesting residual peroxisomal function despite combined peroxin abnormalities. Although a causal contribution from both variants cannot be established, this case highlights the complexity of genotype interpretation in highly consanguineous populations, where multiple rare homozygous variants may coexist within the same individual and potentially contribute to phenotypic variability.

Clinically, neurodevelopmental delay or regression was universal, while visual abnormalities, hearing impairment, gait disturbance, pyramidal signs, seizures, hypotonia, and movement disorders were highly prevalent. This neurological profile observed in our cohort is broadly consistent with prior descriptions of ZSD and related peroxisomal disorders [1, 20]. Importantly, several patients initially presented with sensory deficits, gait dysfunction, or atypical leukodystrophy patterns prior to progression toward more generalized neurological impairment. Notable examples included patients harboring the recurrent *PEX1* p.Gly786Asp variant who presented with Heimler syndrome and predominantly auditory and visual manifestations, as well as a patient with *PEX16* p.Arg287Cys who survived into later childhood with a relatively attenuated phenotype characterized by neurodevelopmental impairment, hearing loss, gait disturbance, movement disorder, pyramidal signs, and microcephaly. Together with the previously reported UAE family, our observation supports an association with prolonged survival [17]. Similarly, patients with *HSD17B4*-related disease often presented with progressive gait dysfunction and spasticity, whereas *ACOX1* deficiency frequently manifested as an inflammatory-appearing leukodystrophy with progressive white matter abnormalities. These observations emphasize that peroxisomal disorders may remain clinically unrecognized outside classical neonatal Zellweger presentations and support the early incorporation of comprehensive genomic testing into the diagnostic evaluation of children with unexplained neurodevelopmental regression, leukodystrophy, sensory impairment, cerebellar dysfunction, or atypical neuroimaging findings.

Although limited by small subgroup sizes, comparison between PBDs and non-PBD peroxisomal disorders revealed substantial clinical overlap despite differences in the underlying molecular mechanisms. Patients with PBDs tended to present earlier and accounted for all mortality within the cohort, reflecting the severe end of the Zellweger spectrum. In contrast, pyramidal signs and seizures were more frequently observed among patients with non-PBD peroxisomal disorders, particularly those with *HSD17B4*-related disease. Although overall severity indices did not differ significantly between groups, PBDs demonstrated a broad phenotypic spectrum ranging from mild Heimler syndrome and infantile Refsum disease to severe neonatal Zellweger presentations. These exploratory findings suggest substantial clinical overlap between PBDs and non-PBD peroxisomal disorders and reinforce the importance of evaluating disorders such as *ACOX1* deficiency, *HSD17B4*-related disease, and *MFF*-related peroxisomal fission disorder alongside classical *PEX*-related disorders in contemporary diagnostic workflows [25, 26].

The proposed severity index was intended as a pragmatic retrospective stratification tool rather than a validated prognostic instrument. Unlike the previously validated 14-domain severity score for Zellweger spectrum disorders, which was developed for prospective assessment and incorporates extensive multisystem manifestations, our simplified index was developed specifically for retrospective multicenter studies and broader applicability across both peroxisome biogenesis disorders and non-PBD peroxisomal disorders [13]. These domains reflect major determinants of neurological disability and disease progression while remaining feasible for routine clinical practice and retrospective chart review. Although patients harboring predicted loss-of-function variants tended to demonstrate higher severity scores than patients with missense variants, these differences did not reach statistical significance. At the same time, several notable exceptions including severe disease associated with *PEX1* p.Gly843Asp and relatively mild disease despite dual *PEX5*/*PEX12* involvement, illustrate the limitations of predicting phenotype solely from variant class. These findings suggest that residual peroxin activity, modifier alleles, metabolic reserve, and environmental factors may all contribute to disease severity. Importantly, patients with major structural or extensive MRI abnormalities demonstrated significantly higher severity indices than patients with normal or mild neuroimaging findings, providing preliminary support for the construct validity of this simplified severity framework. Nevertheless, the severity index was developed specifically for retrospective cohort analysis and should be regarded as an exploratory stratification tool rather than a validated outcome measure. Because the selected domains represent routinely assessed neurological variables, the approach should be readily applicable in similar retrospective cohorts. However, formal evaluation of inter-rater reliability, prospective reproducibility, and direct comparison with the validated Zellweger severity score were beyond the scope of the present study. Future studies involving larger cohorts and external validation across independent populations will be necessary to establish its reproducibility, clinical utility, and prognostic value.

Neuroimaging analysis was particularly informative. MRI abnormalities most commonly involved demyelinating white matter changes, brainstem structures, cerebellar pathways including the dentate hilus and middle cerebellar peduncles, corpus callosum abnormalities, and parieto-occipital white matter involvement. These findings are highly consistent with previously described neuroimaging patterns in peroxisomal disorders [14–16, 27]. Structural cortical abnormalities, including polymicrogyria, were identified in patients with *PEX1*-, *PEX12*-, and *ACOX1*-related disease, all of whom exhibited congenital onset and severe neurological phenotypes within our cohort. This observation is consistent with previous reports linking severe early peroxisomal dysfunction to abnormal cortical development. Serial MRI studies demonstrated recognizable progression patterns across genetically distinct disorders. Patients with *HSD17B4*-related disease demonstrated progressive parieto-occipital and diffuse white matter involvement with cerebellar atrophy, whereas *PEX10*-related disease showed aggressive progression into diffuse white matter, basal ganglia, and brainstem structures. Progressive cerebellar and corpus callosum abnormalities were also observed in *ACOX1*-related disease. Although based on a limited number of longitudinally studied patients, these observations suggest that genetically distinct peroxisomal disorders may share common patterns of neuroanatomical vulnerability despite differences in their underlying molecular defects.

Our findings have important implications for diagnostic practice in high-consanguinity populations. The broad genetic distribution observed in this cohort argues against limited single-gene testing strategies and supports exome-based or comprehensive leukodystrophy-focused genomic approaches. In addition, mild phenotypes such as Heimler syndrome may initially mimic isolated sensory disorders or hereditary ataxias, delaying diagnosis [22]. The high prevalence of rare homozygous variants and variants of uncertain significance further emphasizes the importance of careful phenotypic correlation and the development of population-specific genomic reference datasets for variant interpretation.

### Limitations

Several limitations should be considered. The retrospective multicenter design resulted in incomplete clinical, biochemical, and longitudinal neuroimaging data for some patients, as biochemical investigations, including very-long-chain fatty acid profiles and other peroxisomal biomarkers, were not uniformly available across participating centers. Additionally, MRI protocols were not standardized across centers. Functional validation was unavailable for most novel variants, and many therefore remained classified as VUS despite supportive segregation and phenotype data. Consequently, genotype–phenotype correlations involving these variants should be interpreted cautiously until additional molecular or functional evidence becomes available. The proposed severity index was designed as a pragmatic retrospective phenotypic stratification tool and has not undergone formal prospective validation or assessment of inter-rater reliability. Future multicenter studies comparing this simplified index with the validated Zellweger severity score will be necessary to establish reproducibility, external validity, and prognostic performance. In addition, subgroup sizes for individual genes and disease categories were limited, thereby reducing statistical power for comparative analyses and limiting robust genotype–phenotype inference. Finally, although several recurrent variants were identified in unrelated families, the absence of haplotype analysis precludes confirmation of founder effects. Future population-based genetic studies will be necessary to distinguish recurrent mutations from true founder alleles.

## Supplementary Information

Below is the link to the electronic supplementary material.

Supplementary Material 1: Supplementary Figure S1. Distribution of causative genes across the study cohort. (A) Gene distribution among all genetically identified patients, including non-classical peroxisome-associated disorders. (B) Gene distribution in the primary analysis cohort after exclusion of non-classical phenotypes. PEX1 was the most frequently affected gene

Supplementary Material 2: Supplementary Figure S2. Geographic distribution of participating referral centers across Iran. Map illustrating the locations of the five Iranian referral centers contributing genetically confirmed peroxisomal disorder cases to this multicenter cohort

## Data Availability

All data supporting the findings of this study are available within the paper and its Supplementary Information.
